# Familiarity and genetic literacy among medical students in Indonesia

**DOI:** 10.1186/s12909-021-02946-8

**Published:** 2021-10-09

**Authors:** Yolanda Marthia Swandayani, Ferdy Kurniawan Cayami, Tri Indah Winarni, Agustini Utari

**Affiliations:** 1 Faculty of Medicine, Diponegoro University, Semarang, Indonesia; 2 Department of Anatomy, Faculty of Medicine, Diponegoro University, Semarang, Indonesia; 3 Center for Biomedical Research (CEBIOR), Faculty of Medicine, Diponegoro University, Jl. Prof H. Soedarto, SH, 50275 Semarang, Indonesia; 4 Department of Pediatrics, Faculty of Medicine, Diponegoro University, Semarang, Indonesia

**Keywords:** Genetic literacy, genetic familiarity, medical student, REAL-G

## Abstract

**Background:**

There is a lack of genetic knowledge among health care professionals especially in some developing countries such as Indonesia. Based on our experience, genetic disorders receive less attention in medical education and professionals. This study aims to determine the familiarity and literacy of genetics among medical students in Indonesia.

**Methods:**

A total of 1003 Indonesian medical (pre-clinical and clinical) students completed the Rapid Estimate of Adult Literacy in Genetics (REAL-G) questionnaire with a total score of seven for familiarity and eight for genetic literacy. The Mann-Whitney U test was used to compare the familiarity and genetic literacy scores between pre-clinical and clinical students.

**Results:**

The average scores of familiarity and genetic literacy were 5.63 **±** 0.96 and 6.37 **±** 0.83, respectively. Genetic familiarity was higher (*p* = 0.043) among clinical students than pre-clinical students, while there was no significant difference in genetic literacy (*p* = 0.362) between pre-clinical and clinical students. Genetic familiarity does not impact the level of genetic literacy. However, medical students’ genetic literacy is influenced by demographic characteristics, such as age, sex, university type, genetic learning experience, university accreditation, and university location.

**Conclusions:**

In general, Indonesian medical students have relatively good familiarity and literacy in genetics although further study is necessary to accurately measure the genetic familiarity and literacy in medical students and general public.

## Background

Genetic and genomic technologies have been rapidly developing in low- and middle-income countries in the Asia-Pacific region [1]. This development affects genetic-related health services such as ordering genetic testing, confirming a diagnosis, providing genetic counseling, making a risk assessment, and offering treatment options. The growing need for medical genetic and genomic technology globally demands qualified human resources to support the services. However, there is a gap caused by the lack of professional knowledge, training, medical genetics, clinical genetics, and genetic counseling, especially in Asia [2].

Genetic services in Indonesia are relatively challenging, with limited facilities and expertise [1, 3]. Another obstacle in developing genetic services in Indonesia is the national policy, which prioritizes infant and maternal mortality rates, stunting, tuberculosis, complete primary immunization, and non-communicable diseases such as coronary heart disease, diabetes, hypertension, and cancer [4]. Hitherto, genetic services are rarely considered among the priorities in Indonesian health services. In 2016, the Indonesian Society of Human Genetics was established to facilitate and support genetic science, profession, and others who have a particular interest in Indonesia, including genetic counselors, clinical geneticists, and molecular geneticists, while endeavoring the recognition from the government. Only a few institutions, including hospitals, provide genetic services because genetic diseases are considered incurable [5]. Currently, some private laboratories are offering genetic testing such as prenatal testing, paternity test, inherited cancer panels, and frequent inherited diseases without support from the clinician to provide proper education for patients, as the genetic profession has not been formally acknowledged and registered by the Indonesian Medical Council and Ministry of Health. Thus, genetic literacy is critical, especially among medical professionals who closely deal with individuals with positive genetic results.

Genetic literacy is defined as sufficient knowledge and understanding of genetic principles that can be measured from the familiarity of genetic terminology, clinical skills, and factual knowledge about genes and hereditary traits [6]. Genetic literacy is necessary for medical professionals, especially for the physician, to identify the genetic diseases; thus, patients can receive appropriate management, including counseling. Medical School is the primary education program to obtain clinical genetic knowledge [7]. However, previous studies have reported that medical schools are inadequately preparing future physicians with a sufficient understanding of genetic literacy [8–11]. Less familiarity and genetic literacy among physicians may result in misdiagnosis, treatment failure, and higher genomic testing, which is not needed [12, 13]. This is the first study to assess the familiarity and genetic literacy among medical students in Indonesia.

## Methods

**Fig. 1:**
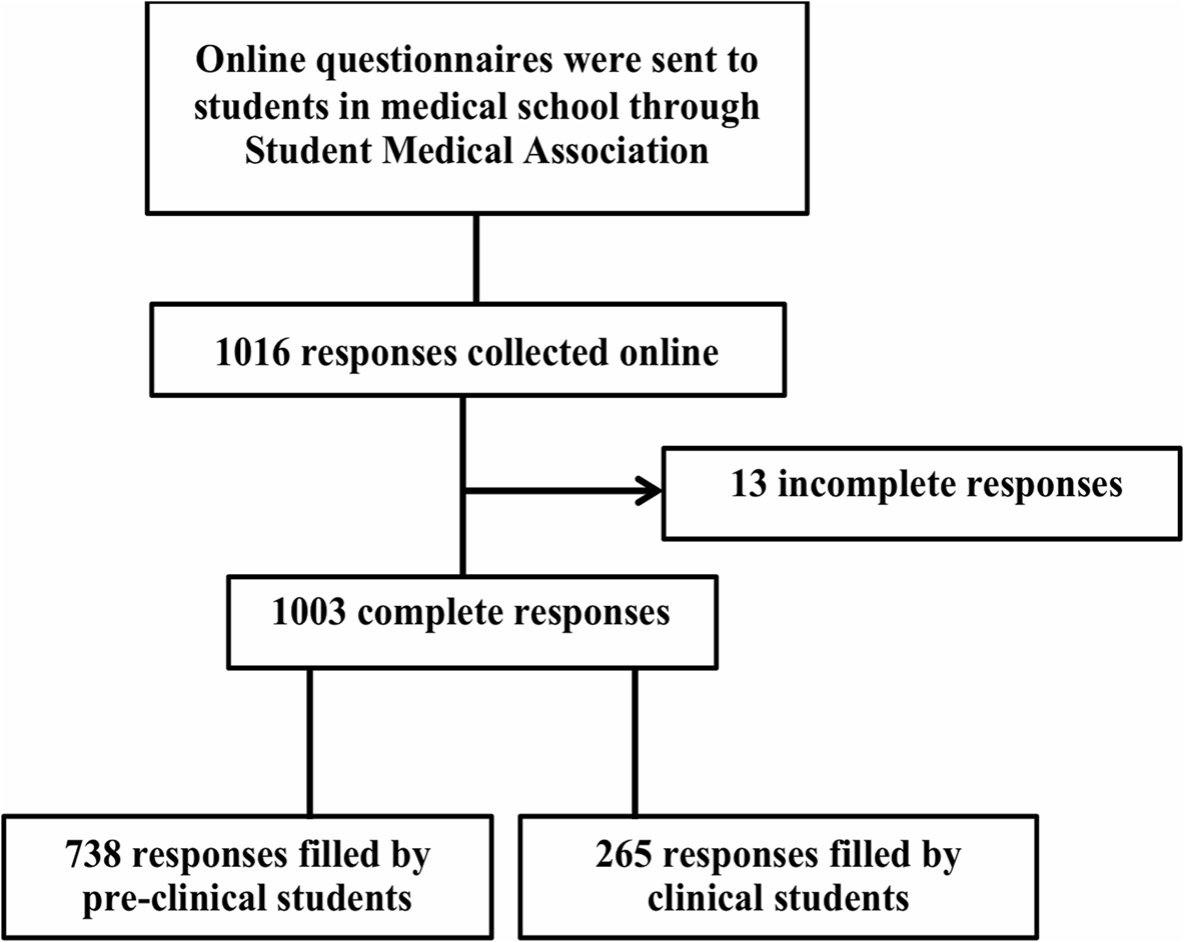
Flow chart of study participants

This study was a cross-sectional study with a consecutive sampling method. An online questionnaire was shared with medical students in Indonesia through the Indonesian Medical Student Association. A total of 1016 medical students from 47 universities in Indonesia filled an electronic survey between May and September 2019 however, a total of 13 questionnaire were incomplete. Total 1003 respondents were grouped into pre-clinical and clinical students based on the current medical education system in Indonesia. Detailed is shown in Fig. 1. Pre-clinical students were undergraduate students in medical school before entering the clerkship. Conversely, clinical students were students in clinical training rotation for two years following the pre-clinical stage [14]. This study was approved by the Health Research Ethics Committee, Faculty of Medicine, Diponegoro University No.201/EC/KEPK/FK-UNDIP/V/2019. All respondents gave informed consent electronically before enrollment in this study. A preliminary survey to validate our online questionnaire was conducted online and paper-based by 50 medical students from Diponegoro University, Semarang, Indonesia.

The the familiarity and genetic literacy in our study were assessed using the Rapid Estimate of Adult Literacy in Genetics (REAL-G) validated in a previous studies [15–17]. The questionnaire was translated from English into Bahasa Indonesia and back translated to English by an authorized translator. Demographic information such as age, sex, family history of genetic diseases, genetic learning experience, university accreditation, and university location was obtained before the REAL-G questionnaire. University accreditation was classified as grade A as the highest rank, grade B as a middle rank, and grade C as the lowest rank determined by an independent institution authorized to assess and determine the grade of the university study program.

REAL-G consisted of 8 genetic terms, that are, genetic, chromosome, vulnerability, mutation, variation, abnormality, hereditary, and sporadic. The medical students’ level of familiarity was measured using a seven-point Likert-scale, ranging from ‘very unfamiliar’ (1), ‘moderately unfamiliar’ (2), ‘slightly unfamiliar’ (3), ‘familiar’ (4), ‘slightly familiar’ (5), ‘moderately familiar’ (6), and ‘very familiar’ (7). The score was based on respondents’ average perceived familiarity across the eight terms. A total score of correct answers of eight multiple-choice questions related to the terms was obtained to measure the genetic literacy, with a maximum score of eight and a minimum score of zero. The questionnaire is provided as a supplementary file.

Analysis was performed using the SPSS v.25 for Windows. Pearson’s chi-squared test was used to compare the variables between pre-clinical and clinical students. We used the Kolmogorov-Smirnov test to analyze the normality of the distribution of the numeric data. The Mann-Whitney U test was conducted to compare the familiarity and genetic literacy scores between pre-clinical and clinical students. Kendall’s Tau test measured the correlation between familiarity and genetic literacy. Factors affecting each term of genetic literacy were determined using the regression analysis. The variables included in the regression analysis are the previously variables analyzed by bivariate analysis and resulted as p-value < 0.25.

## Results

This study included 1003 students of 738 pre-clinical and 265 clinical students. The demographic information of pre-clinical and clinical students is displayed in Table 1. Respondents’ ages ranged from 16 to 28 years, with an average age of 20.6 years, and the majority of respondents were female (67.7 %). The majority of the respondents studied at the public, grade-“A”-accredited university, and the universities were mostly located on Java Island.

**Table 1:** Demographic characteristic of respondents

Variables	Familiarity		Genetic Literacy	
	(Average ± SD)	P	(Average ± SD)	P
Age				
- ≤ 21 (n = 765)	5.61 **±** 0.94	0.090^‡^	6.39 **±** 0.82	0.198^‡^
- > 21 (n = 238)	5.71 **±** 0.99		6.31 **±** 0.85	
Gender				
- Male (n = 324)	5.74 ± 0.907	0.037^‡*^	6.35 ± 0.866	0.820^‡^
- Female (n = 679)	5.58 ± 0.974		6.38 ± 0.806	
Type of university				
- Public (n = 831)	5.66 ± 0.953	0.043^‡*^	6.40 ± 0.829	0.002^‡*^
- Private (n = 172)	5.50 ± 0.960		6.22 ± 0.793	
Family history of genetic diseases				
- Yes (n = 62)	5.61 ± 0.997	0.919^‡^	6.42 ± 0.801	0.805^‡^
- No (n = 941)	5.63 ± 0.953		6.37 ± 0.827	
Genetic learning experience				
- Yes (n = 935)	5.65 ± 0.952	0.044^‡*^	6.38 ± 0.826	0.369^‡^
- No (n = 68)	5.41 ± 0.982		6.29 ± 0.811	
University Accreditation				
- A (n = 691)	5.71 ± 0.941	0.001^”*^	6.40 ± 0.805	0.327^”^
- B (n = 110)	5.55 ± 0.986		6.32 ± 0.753	
- C (n = 202)	5.42 ± 0.959		6.32 ± 0.924	
Location of University				
- Java (n = 708)	5.61 ± 0.961	0.275^‡^	6.35 ± 0.802	0.045^‡*^
- Outside Java (n = 295)	5.68 ± 0.941		6.44 ± 0.881	

* Significant (p<0.05)

‡ Mann Whitney

“*Kruskal−Wallis*

### Genetic Familiarity

On average, the respondents’ familiarity total score was 5.63 ± 0.96 out of 7. As shown in Table 2, male respondents (5.74 ± 0.907) had a significantly higher average genetic familiarity score than female respondents (5.58 ± 0.974) (p = 0.037). Students with genetic learning experience scored significantly higher on genetic familiarity assessment (5.65 ± 0.952; p = 0.044). In addition, students from public (5.66 ± 0.953; p = 0.043) and grade-“A”-accredited universities (5.71 ± 0.941; p = 0.001) also had significantly higher scores for genetic familiarity than other students.

**Table 2:** Factors affecting familiarity and genetic literacy among medical students

	Pre-clinical	Clinical	P
Familiarity (1–7)	5.60 ± 0.96	5.73 ± 0.94	0.043^‡*^
Genetic Literacy (1–8)	6.38 ± 0.84	6.34 ± 0.78	0.362^‡^
Mean of Total Score	5.63 ± 0.96	6.37 ± 0.83	

* Significant (*p*<0.05)

‡ Mann Whitney

Table 3 shows that the average score for genetic familiarity was higher in clinical students (5.73 ± 0.94) than pre-clinical students (5.60 ± 0.96, p = 0.043). Furthermore, we have separately analyzed the genetic familiarity score between pre-clinical and clinical students based on the demographic variables. As displayed in Table 4, significant differences in pre-clinical familiarity scores are seen in university type, university accreditation, and genetic learning experience variables. Students from public (5.65 **±** 0.95, p = 0.003), grade-“A”-accredited universities (5.71 **±** 0.93, p = 0.001), and students with genetic learning experience (5.62 **±** 0.96, p = 0.016) had higher score of genetic familiarity than other students. While in clinical students, the genetic familiarity score was significantly higher in the students from the universities outside of Java Island (6.08 **±** 0.70, p = 0.002) than the universities in Java Island.

**Table 3:** Comparison of familiarity and genetic literacy scores between pre-clinical and clinical students

Variables	Familiarity				Genetic Literacy				
	Pre-clinical		Clinical		Pre-clinical		Clinical		
	(Average ± SD)	P	(Average ± SD)	P	(Average ± SD)	P	(Average±SD)	P	
Age									
- ≤ 21 (n = 765)	5.59 **±** 0.94 (n = 684)	0.455^‡^	5.75 **±** 0.91 (n = 81)	0.971^‡^	6.40 **±** 0.83 (n = 684)	0.041^‡*^	6.31 **±** 0.72 (n = 81)	0.480^‡^	
- > 21 (n = 238)	5.64 **±** 1.15 (n = 54)		5.73 **±** 0.95 (n = 184)		6.17 **±** 0.97 (n = 54)		6.35 **±** 0.81 (n = 184)		
Gender									
- Male (n = 324)	5.71 **±** 0.91 (n = 218)	0.067^‡^	5.80 **±** 0.89 (n = 106)	0.473^‡^	6.36 **±** 0.89 (n = 218)	0.908^‡^	6.34 **±** 0.82 (n = 106)	0.927^‡^	
- Female (n = 679)	5.55 **±** 0.98 (n = 520)		5.69 ± 0.97 (n = 159)		6.39 **±** 0.82 (n = 520)		6.34 ± 0.76 (n = 159)		
Type of university									
- Public (n = 831)	5.65 **±** 0.95 (n = 606)	0.003^‡*^	5.70 **±** 0.96 (n = 225)	0.184^‡^	6.41 **±** 0.85 (n = 606)	0.018^‡*^	6.38 **±** 0.78 (n = 225)	0.033^‡*^	
- Private (n = 172)	5.37 **±** 0.97 (n = 132)		5.93 **±** 0.80 (n = 40)		6.25 **±** 0.80 (n = 132)		6.13 **±** 0.76 (n = 40)		
Family history of genetic diseases									
- Yes (n = 62)	5.49 **±** 1.02 (n = 46)	0.617^‡^	5.96 **±** 0.85 (n = 16)	0.348^‡^	6.43 **±** 0.86 (n = 46)	0.743^‡^	6.38 **±** 0.62 (n = 16)	0.944^‡^	
- No (n = 941)	5.60 **±** 0.96 (n = 692)		5.72 **±** 0.94 (n = 249)		6.38 **±** 0.84 (n = 692)		6.34 **±** 0.79 (n = 249)		
Genetic learning experience									
- Yes (n = 935)	5.62 **±** 0.96 (n = 693)	0.016^‡*^	5.74 **±** 0.93 (n = 242)	0.762^‡^	6.39 **±** 0.84 (n = 693)	0.535^‡^	6.35 **±** 0.78 (n = 242)	0.527^‡^	
- No (n = 68)	5.28 **±** 0.95 (n = 45)		5.67 **±** 1.02 (n = 23)		6.31 **±** 0.79 (n = 45)		6.26 **±** 0.86 (n = 23)		
University Accreditation									
- A (n = 691)	5.71 **±** 0.93 (n = 473)		5.71 **±** 0.96 (n = 218)	0.323^”^	6.41 ± 0.82 (n = 473)	0.457^”^	6.37 **±** 0.77 (n = 218)	0.132^”^	
- B (n = 110)	5.46 **±** 1.02 (n = 76)	0.001^”*^	5.77 **±** 0.88 (n = 34)		6.39 ± 0.77 (n = 76)		6.15 **±** 0.70 (n = 34)		
- C (n = 202)	5.38 **±** 0.96 (n = 189)		6.12 **±** 0.71 (n = 13)		6.32 ± 0.91 (n = 189)		6.31 ± 1.18 (n = 13)		
Location of University									
- Java (n = 708)	5.59 **±** 0.96 (n = 501)	0.824^‡^	5.64 **±** 0.97 (n = 207)	0.002^‡*^	6.36 **±** 0.81 (n = 501)	0.150^‡^	6.34 **±** 0.76 (n = 207)	0.642^‡^	
- Outside Java (n = 295)	5.61 **±** 0.97 (n = 237)		6.09 **±** 0.70 (n = 58)		6.43 **±** 0.91 (n = 237)		6.33 **±** 0.87 (n = 58)		

* Significant (p<0.05)

‡ Mann Whitney

“*Kruskal−Wallis*

**Table 4:** REAL-G familiarity and genetic literacy scores between pre-clinical and clinical students based on the demographic variables

	Familiarity (n = 1003) average ± SD			Genetic Literacy (n = 1003) (% correct answer)		
	**Pre-clinical**	**Clinical**	**p**	**Pre-clinical**	**Clinical**	**P**
Term 1 :	5.72 ± 1.185	5.72 ± 1.117	0.817^‡^	81.57 %	68.68 %	< 0.001^¥*^
Genetic						
Term 2 :	5.90 ± 1.09	5.89 ± 1.062	0.670^‡^	95.26 %	94.34 %	0.556^¥^
Chromosome						
Term 3 :	5.50 ± 1.255	5.63 ± 1.181	0.356^‡^	87.40 %	90.57 %	0.170^¥^
Vulnerability						
Term 4 :	5.91 ± 1.059	5.96 ± 1.023	0.414^‡^	99.86 %	100 %	0.549^¥^
Mutation						
Term 5 :	5.40 ± 1.212	5.55 ± 1.205	0.066^‡^	6.78 %	4.15 %	0.125^¥^
Variation						
Term 6 :	5.96 ± 1.073	5.95 ± 1.038	0.563^‡^	98.78 %	98.50 %	0.720^¥^
Abnormality						
Term 7 :	6.06 ± 1.042	6.06 ± 0.979	0.093^‡^	99.60 %	99.62 %	0.948^¥^
Hereditary						
Term 8 :	4.30 ± 1.750	5.11 ± 1.418	< 0.001^‡*^	69.24 %	78.11 %	0.006^¥*^
Sporadic						
Mean of Total Score	5.60 ± 0.96	5.73 ± 0.94	0.043^‡*^	79.8 %	79.2 %	0.579^¥^

* Significant (p<0.05)

‡ Mann Whitney

¥ Pearson chi square

SD standard deviation

Both pre-clinical students (6.06 ± 1.042) and clinical students (6.06 ± 0.979) were more familiar with the term “hereditary” compared to other terms. In general, both groups were also least familiar with the term “sporadic” while pre-clinical students (4.30 ± 1.750) were significantly less familiar compared to clinical students (5.11 ± 1.418, p < 0.001). Although both groups were least familiar with the term “sporadic,” more than half of the students answered the term “sporadic” correctly. Only a small number of students in both groups (6.78 % in the pre-clinical group and 4.15 % in the clinical group) could correctly answer the question term “variation” although most of the students admitted their familiarity with the term “variation”.

### Genetic Literacy

The average total score of genetic literacy was 6.37 **±** 0.83 (range, 1–8). Genetic literacy score was higher in students from universities outside Java Island (6.44 ± 0.881, p = 0.002) than students from the universities in Java Island. Furthermore, students from public universities (6.40 ± 0.829, p = 0.002) also had a significantly higher level of genetic literacy than private universities. (See Table 2)

Table 3 shows no significant difference in the average score of genetic literacy between pre-clinical (6.38 ± 0.84) and clinical students (6.34 ± 0.78, p = 0.362). Despite this, both pre-clinical (6.41 **±** 0.85, p = 0.018) and clinical students (6.38 **±** 0.78, p = 0.033) from public universities scored significantly higher on genetic literacy assessment. Pre-clinical students younger than or 21 years old (6.40 **±** 0.83, p = 0.041) also had a significantly higher genetic literacy than those older than 21 years (see Table 4).

Almost all students answered the question about term “mutation” correctly, but most of them were unable to answer the term “variation” However, the familiarity score for term “variation” were high. Further analysis comparing the differences between pre-clinical and clinical group found that there were significant differences in the terms “genetic” (< 0.001) and “sporadic” (p = 0.006) (See Table 5).

**Table 5:** The average familiarity and the percentage of correct answer of genetic literacy scores among pre-clinical and clinical medical students

	Pre-clinical (p)	Clinical (p)	All respondents (p)
Term 1 : Genetic	0.003^*^	0.239	0.004^*^
Term 2 : Chromosome	0.151	0.494	0.193
Term 3 : Vulnerability	0.287	0.231	0.414
Term 4 : Mutation	0.123	< 0.001^*^	0.125
Term 5 : Variation	0.490	0.429	0.426
Term 6 : Abnormality	0.438	0.147	0.330
Term 7 : Hereditary	0.034^*^	0.045^*^	0.008^*^
Term 8 : Sporadic	0.028^*^	0.311	0.003^*^
Total Score	0.263	0.237	0.192

Analysis using Kendall’s Tau Test

* Significant (p<0.05)

### Correlation between familiarity and genetic literacy

Based on the results, a positive correlation (r = 0.22) was found between familiarity and genetic literacy scores. However, the result was not significant (p = 0.192). In general, there was no association between familiarity and genetic literacy scores. Then, we separately analyzed the correlation between familiarity and genetic literacy scores for each term. Table 6 shows that there was a significant correlation between familiarity and genetic literacy scores in three terms (p < 0.05), that are, term number 1 (“genetic”, p = 0.004), term number 7 (“hereditary”, p = 0.008), and term number 8 (“sporadic”, p = 0.003). Furthermore, we analyzed the correlation of each term in the familiarity and genetic literacy questionnaire separately for pre-clinical and clinical students. In pre-clinical students, there was a significant correlation in three terms (p < 0.05), i.e., term number 1 (“genetic”, p = 0.003), term number 7 (“hereditary”, p = 0.034), and term number 8 (“sporadic”, p = 0.028). While, in clinical students, there was a significant correlation in term number 4 (“mutation”, p = < 0.001) and term number 7 (“hereditary”, p = 0.045) (see Table 6).

**Table 6:** Correlation between familiarity and genetic literacy in each term

	Variable	B	p	OR	95 % CI
Term 1 : Genetic	Age	-0.393	0.024^*^	0.675	0.481–0.949
	University Accreditation	0.191	0.090	1.210	0.971–1.509
	Location of University	0.500	0.011^*^	1.649	1.119–2.431
	Familiarity	0.179	0.006^*^	1.196	1.053–1.359
Term 2 : Chromosome	Gender	-0.550	0.114	0.577	0.291–1.141
Term 3 : Vulnerability	University Accreditation	-0.402	< 0.001^*^	0.669	0.539–0.830
Term 4 : Mutation	Familiarity	-13.739	0.989	0.000	0.653–1.396
Term 5 : Variation	University Accreditation	0.437	0.003^*^	1.548	1.164–2.059
Term 6 : Abnormality	Location of University	-1.045	0.062	0.352	0.117–1.055
Term 7 : Hereditary	Familiarity	0.836	0.014^*^	2.308	1.183–4.503
Term 8 : Sporadic	Type of University	-0.362	0.066	0.697	0.474–1.024
	University Accreditation	-0.170	0.074	0.844	0.700-1.016
	Familiarity	0.750	0,073	1,078	0.993–1.171

Analysis using binomial regression test

B: Regression coefficient (positive coefficient means positive correlation between dependent and independent variables and vice versa)

*Significant (p < 0.05)

OR: Odds Ratio (measure the strength of association between dependent and independent variables)

### Factors affecting genetic literacy scores

Based on the results of the binomial regression test, the first term (genetic) was significantly affected by age (p = 0.024, OR = 0.675, B = -0.393), location of university (p = 0.011, OR = 1.649, B = 0.500), and familiarity (p = 0.006, OR = 1.196, B = 0.179). The university location was the most dominant variable. Students from universities outside of Java Island, younger than or age 21, and students with higher familiarity scores of the “genetic” term could answer correctly. The third (“vulnerability”, p < 0.001) and fifth term (“variation”, p = 0.003) were significantly influenced by the university accreditation. Students from grade-“A”-accredited university could answer the term “vulnerability” correctly (OR = 0.669, B = -0.402). However, students from grade-“C”-accredited university were more likely to answer the question about “variation” correctly (OR = 1.548, B = 0.437). The seventh term (“hereditary”) was significantly affected by familiarity (p = 0.014). Students with higher familiarity score of term “hereditary” could answer correctly (OR = 2.308, B = 0.836). However, there was no significant effect of variables on terms number 2 (chromosome), 4 (mutation), 6 (abnormality), and 8 (sporadic) (see Table 7).

**Table 7:** Factors Affecting Genetic Literacy Scores of Each Question on REAL-G

Variables	Pre-clinical (n = 738)	Clinical (n = 265)	P
	Frequency (n%)	Frequency (n%)	
Age (Average **±** SD, min-max)	20.00 ± 1.19 (16–25)	22.21 ± 1.42 (19–28)	< 0.001^‡*^
Gender			
- Male (n = 324)	218 (29.5 %)	106 (40.0 %)	0.002^¥*^
- Female (n = 679)	520 (70.5 %)	159 (60 %)	
Type of university			
- Public (n = 831)	606 (82.1 %)	225 (84.9 %)	0.092^¥^
- Private (n = 172)	132 (17.9 %)	40 (15.1 %)	
Family history of genetic diseases			
- Yes (n = 62)	46 (6.2 %)	16 (6.0 %)	0.910^¥^
- No (n = 941)	692 (93.8 %)	249 (94.0 %)	
Genetic learning experience			
- Yes (n = 935)	693 (93.9 %)	242 (91.3 %)	0.152^¥^
- No (n = 68)	45 (6.1 %)	23 (8.7 %)	
University Accreditation			
- A (n = 691)	473 (64.1 %)	218 (82.3 %)	< 0.001^¥*^
- B (n = 110)	76 (10.3 %)	34 (12.8 %)	
- C (n = 202)	189 (25.6 %)	13 (4.9 %)	
Location of University			
- Java (n = 708)	501 (67.9 %)	207 (78.1 %)	0.002^¥*^
- Outside Java (n = 295)	237 (32.1 %)	58 (21.9 %)	

*Significant (p < 0.05)

‡Mann Whitney

¥ Pearson chi square

SD : standard deviation

## Discussion

As far as our concern, there is no study on familiarity and genetic literacy in the Southeast Asia region, especially in Indonesia. Unexpectedly, the familiarity and genetic literacy scores from our study were relatively high with the REAL-G questionnaire compared to American population.[6, 15] As the REAL-G questionnaire was not applied in study with health professional as respondents, we compared our results from different studies with other tools such as Genomic Nursing Concept Inventory (GNCI) or other self-made questionnaire for nutritionist. [9, 10] In general, our respondents had higher genetic literacy score although there is limited genetic service even in tertiary hospitals in Indonesia. Furthermore, genetics in Indonesia is taught to medical students in related clinical fields, such as pediatric. Given this background, we assumed that the familiarity and genetic literacy of medical students in Indonesia is relatively similar to that of the general public in developed countries where the tool was established.

The higher familiarity and genetic literacy score in our respondents may be attributed by the admission system which only allows high school students majoring in natural sciences to apply for medical school either in public or private university, thus, majority of medical students are more intelligent and confident. There was a significant difference in the genetic familiarity score between pre-clinical and clinical students. Clinical students had higher familiarity scores than pre-clinical students. Still, there was no significant difference in genetic literacy, which may be due to clinical students having a higher level of confidence than pre-clinical students. Previous studies concluded that self-confidence has a significant influence on the learning process. Self-confidence students had better academic achievement [18–20].

Additionally, clinical students had more exposures and more experiences than pre-clinical students, and those who have more opportunities to deal with genetic patients will have higher familiarity perception. Previous studies also showed that a higher level of education also increases students’ level of self-confidence and knowledge [8, 21]. Factors that affect familiarity were genetic learning experiences, sex, university accreditation, and university type, while age, type, and location of university affect genetic literacy. Having learning experiences about genetics affected genetic familiarity score significantly but did not affect genetic literacy score. Similar results were obtained in the pre-clinical group, where students with genetic learning experiences had significantly higher genetic familiarity score than those without genetic learning experiences. This finding was supported by a study revealing that students who participated in genetic courses had significantly higher genetic knowledge [9].

This study also evaluated the difference between familiarity and genetic literacy scores among students from different university’s accreditation levels. Students from universities with grade-“A”-accreditation had higher familiarity scores than others; however, there was no significant difference in the genetic literacy score. In the pre-clinical group, students from grade-“A”-accredited universities had significantly higher familiarity scores than students from grade-“B and C”-accredited university. A study from Vietnam showed that accreditation significantly improves the university’s quality of teaching, learning research, and management [22]. Accreditation also influenced students’ decisions to choose their study programs, implying that students with higher educational scores and abilities would undoubtedly select a study program with higher or best accreditation grades [23].

In general, students in public universities had significantly higher average scores in both familiarity and genetic literacy score compared to students from private universities (Table 2). However, further analysis showed that students from public universities in the clinical group had lower familiarity scores than students from private universities (Table 4). It may be due to a small number of students from private universities in the clinical group. The high familiarity and genetic literacy scores of students from public universities in our study may have been influenced by the popularity of public universities in Indonesia, where the competitive selection was applied in the study enrollment program [23]. As a result, students from public university are generally more motivated than students from private university.

Our results revealed that respondents were significantly more familiar with the term “hereditary” and less familiar with the term “sporadic” than other terms. Another study in general population have reported that respondents were more familiar with the term “hereditary” and least familiar with the term “sporadic” [6]. This might be because hereditary diseases were often shown as topics either on television or on social media, thus, term “hereditary” is a more common term in general public than other genetic terms from our questionnaire.

Interestingly, most students were unable to answer questions about the term “variation” correctly, but their familiarity was still high. On the other hand, familiarity in the term “sporadic” was the lowest, although it was not the term with the lowest literacy score. These interesting results may be caused by the use of term “variation” and “sporadic,” which has more specific meaning in genetic terms than the medical terms. Furthermore, medical students in Indonesian are generally exposed to genetic subjects indirectly through other clinical fields. This result needs to be addressed as the familiarity without proper literacy may cause incorrect education to the patients. As a result, improper management and understanding may endanger the safety of patients with genetic disease as the background. Thus, appropriate genetics subject is necessary to be included in curricula and competency in medical education to ensure holistic treatment provided for the patients.

In general, our study found no correlation between familiarity and genetic literacy total score. However, significant correlations between familiarity and genetic literacy in total sample were found in “genetic”, “hereditary,” and “sporadic” terms. Similar results were obtained in the pre-clinical group, whereas significant correlations in the clinical group were only found in “mutation” and “hereditary” term. Further analyses found some factors turned out to affect the genetic literacy score for each question and familiarity at term “genetic”; university accreditation at term “vulnerability” and “variation”; and familiarity at term “hereditary”. These results showed that genetic literacy is influenced by various factors such as age, sex, university type, genetic learning experience, university accreditation, and location of the university.

## Study limitations

It is difficult to approach all the students directly from all universities, as there is no centralized data in Indonesia, so the online questionnaire was distributed through medical student associations. Consequently, it was difficult to determine the response rate for this study. Furthermore, the REAL-G questionnaire might not be ideal for medical students, who are better educated than the general public, resulting in higher genetic literacy scores compared to other studies.

## Conclusions

The findings indicate that the familiarity and genetic literacy of Indonesian medical students is relatively high. There was no significant difference between pre-clinical and clinical students in the REAL-G scores. However, as this is the first study conducted in Indonesia, further study is necessary to accurately evaluate the familiarity and genetic literacy of Indonesian medical students. Further study is necessary using more elaborate tools such as the Genetic Literacy Assessment Instrument (GLAI) to evaluate the genetic literacy of medical students in Indonesia with a more systematic collection of respondents.

## Data Availability

The datasets used and/or analyzed during the current study are available from the corresponding author on reasonable request.

## Abbreviations

REAL-G: Rapid Estimate of Adult Literacy in Genetics
GLAI: Genetic Literacy Assessment Instrument

## References

[1] Thong MK, See-Toh Y, Hassan J, Ali J (2018). Medical genetics in developing countries in the Asia-Pacific region: challenges and opportunities. Genet Med.

[2] Laurino MY, Sternen DL, Thompson JK, Leppig KA (2017). Identifying opportunities for collaboration and growth of genetic counseling services in the Asia Region. J Community Genet.

[3] Laurino MY, Leppig KA, Abad PJ, Cham B, Chu YWY, Kejriwal S (2018). A Report on Ten Asia Pacific Countries on Current Status and Future Directions of the Genetic Counseling Profession: The Establishment of the Professional Society of Genetic Counselors in Asia. J Genet Couns.

[4] KEMENKES-RI. Lima Isu Prioritas, Tantangan Badan Peneliti dan Pengembangan Kesehatan 5 Tahun ke Depan. https://www.kemkes.go.id (2019). Accessed 20 Dec 2019.

[5] Ariani Y, Soeharso P, Sjarif DR (2017). Genetics and genomic medicine in Indonesia. Mol Genet Genomic Med.

[6] Abrams LR, McBride CM, Hooker GW, Cappella JN, Koehly LM (2015). The Many Facets of Genetic Literacy: Assessing the Scalability of Multiple Measures for Broad Use in Survey Research. PloS one.

[7] Plunkett-Rondeau J, Hyland K, Dasgupta S (2015). Training future physicians in the era of genomic medicine: trends in undergraduate medical genetics education. Genet Med.

[8] Chow-White P, Ha D, Laskin J (2017). Knowledge, attitudes, and values among physicians working with clinical genomics: a survey of medical oncologists. Hum Resour Health.

[9] Wilkins JG. Knowledge and Perception of College Students Toward Genetic Testing for Personalized Nutrition Care (2017). Accessed 5 Oct 2019.

[10] Wright H, Zhao L, Birks M, Mills J (2019). Genomic Literacy of Registered Nurses and Midwives in Australia: A Cross-Sectional Survey. J Nurs Scholarsh.

[11] Adams K, Butsch W, Kohlmeier M. The State of Nutrition Education at US Medical Schools. J Biomed Educ. 2015:1–7. 10.1155/2015/357627.

[12] Collier R (2012). Genetic literacy poor in primary care. Can Med Assoc J.

[13] Christensen KD, Vassy JL, Jamal L, Lehmann LS, Slashinski MJ, Perry DL (2016). Are physicians prepared for whole genome sequencing? a qualitative analysis. Clin Genet.

[14] Mustika R, Nishigori H, Ronokusumo S, Scherpbier A (2019). The Odyssey of Medical Education in Indonesia. Asia Pac Scholar.

[15] Erby LH, Roter D, Larson S, Cho J (2008). The rapid estimate of adult literacy in genetics (REAL-G): a means to assess literacy deficits in the context of genetics. Am J Med Genet A.

[16] Hooker GW, Peay H, Erby L, Bayless T, Biesecker BB, Roter DL (2014). Genetic literacy and patient perceptions of IBD testing utility and disease control: a randomized vignette study of genetic testing. Inflamm Bowel Dis.

[17] Rodríguez SA, Roter DL, Castillo-Salgado C, Hooker GW, Erby LH (2015). Translation and validation of a Spanish-language genetic health literacy screening tool. Health psychology: official journal of the Division of Health Psychology American Psychological Association.

[18] Aryana M (2010). Relationship Between Self-esteem and Academic Achievement Amongst Pre-University Students. J Appl Sci.

[19] Shaukat S, Bashir M (2016). University Students’ Academic Confidence: Comparison between Social Sciences and Natural Science Disciplines. J Elem Educ.

[20] Arshad M, Zaidi SM, Mahmood D (2015). Self-Esteem & Academic Performance among University Students. Journal of Education Practice.

[21] Dodson CH, Lewallen LP (2011). Nursing students’ perceived knowledge and attitude towards genetics. Nurse Educ Today.

[22] Nguyen HC, Ta TTH (2018). Exploring impact of accreditation on higher education in developing countries: a Vietnamese view. TEAM.

[23] Kusumawati A, Yanamandram VK, Perera N. Student Choice Criteria for Selecting an Indonesian Public University: A Preliminary Finding. ANZMAC 2010 Doctoral Colloquium. 2010.

